# Fluid Intake Monitoring System Using a Wearable Inertial Sensor for Fluid Intake Management

**DOI:** 10.3390/s20226682

**Published:** 2020-11-22

**Authors:** Hsiang-Yun Huang, Chia-Yeh Hsieh, Kai-Chun Liu, Steen Jun-Ping Hsu, Chia-Tai Chan

**Affiliations:** 1 Department of Biomedical Engineering, National Yang-Ming University, Taipei 112, Taiwan; huangshoy@ym.edu.tw (H.-Y.H.); g39904006@ym.edu.tw (C.-Y.H.); 2 Research Center for Information Technology Innovation, Academia Sinica, Taipei 115, Taiwan; t22302856@citi.sinica.edu.tw; 3 Department of Information Management, Minghsin University of Science and Technology, Hsinchu County 304, Taiwan; steenhsu@must.edu.tw

**Keywords:** fluid intake monitoring, drinking activity recognition, drinking amount estimation, wearable inertial sensor

## Abstract

Fluid intake is important for people to maintain body fluid homeostasis. Inadequate fluid intake leads to negative health consequences, such as headache, dizziness and urolithiasis. However, people in busy lifestyles usually forget to drink sufficient water and neglect the importance of fluid intake. Fluid intake management is important to assist people in adopting individual drinking behaviors. This work aims to propose a fluid intake monitoring system with a wearable inertial sensor using a hierarchical approach to detect drinking activities, recognize sip gestures and estimate fluid intake amount. Additionally, container-dependent amount estimation models are developed due to the influence of containers on fluid intake amount. The proposed fluid intake monitoring system could achieve 94.42% accuracy, 90.17% sensitivity, and 40.11% mean absolute percentage error (MAPE) for drinking detection, gesture spotting and amount estimation, respectively. Particularly, MAPE of amount estimation is improved approximately 10% compared to the typical approaches. The results have demonstrated the feasibility and the effectiveness of the proposed fluid intake monitoring system.

## 1. Introduction

Fluid intake has great impacts on health and well-being for individuals, including normal people and patients with chronic diseases. Sufficient water is important for people to maintain body fluid homeostasis, which is related to body temperature control, cognition function, kidney function and heart function [1]. However, most normal people in busy lifestyles often forget to drink sufficient water and neglect the importance of fluid intake. Mild dehydration happens to people with sedentary lifestyles and occupations [2], which increases body weight and risk of chronic diseases [3,4]. Furthermore, inadequate fluid intake decreases physical and mental functions [5], and leads to negative health consequences, such as headache, dizziness and urolithiasis [6,7]. Therefore, fluid intake management is critical to assist people to manage individual drinking behaviors.

Several typical approaches are used to understand and establish drinking behaviors and intake information for management of fluid intake [8,9]. These approaches utilize questionnaires to manually record fluid intake events during a normal week. The recorded fluid intake information, including drinking frequency, drinking time and drinking amount, can assist people and clinical professionals to achieve fluid intake management. However, the typical approaches have issues of manual error and are time-consuming [9,10]. With the advancement of micro-electro mechanical system (MEMS) technology, sensor networks and ubiquitous computing are utilized to recognize fluid intake activities automatically and accurately [11,12].

Automatic fluid intake monitoring systems can be divided into two categories: ambient-based and wearable-based systems. First, ambient-based fluid intake monitoring systems utilize cameras installed in environments [13,14] or sensors embedded on containers [15,16,17] to recognize drinking activities. However, ambient-based systems may suffer in some issues, such as confined spaces, privacy issues and inconveniences. Second, wearable-based systems detect fluid intake by placing acoustic [18,19] and inertial sensors [20,21,22,23] on the human body. Among these sensors, wearable inertial sensors are widely employed to recognize fluid intake activities because of advantages including light weight, low cost and unobtrusiveness. However, most fluid intake systems using wearable inertial sensors only focus on drinking activity recognition. Few studies aim to estimate fluid intake amount using wearable inertial sensors [24]. 

The objective of this work is to propose a fluid intake monitoring system with a wearable inertial sensor. The proposed system applies a hierarchical approach to detect drinking activities, recognize drinking gestures and estimate intake amount. Various types of fluid intake information are exploited for fluid intake estimation for the robustness of the proposed system. The main contribution of this work is listed as follows:

- Drinking with different types of containers may affect the performance of fluid intake. It would lead to technical problems of the diversity and variability to fluid intake monitoring, especially to amount estimation. Most previous works did not tackle these problems. Therefore, container-dependent amount estimation models are proposed to enhance the reliability of the fluid intake monitoring system.
- The previous amount estimation approach only used typical statistical approaches (e.g., linear regression) for intake amount assessment. An elaborate approach based on machine learning models should be explored for reliable wearable-based fluid intake estimation. Therefore, this work applies a machine-learning-based estimation approach (e.g., support vector machine regression) to improve the performance on the amount estimation.

The rest of this work is organized as follows: we briefly introduce related works of drinking activity recognition and amount estimation in Section 2. The proposed fluid intake monitoring system is presented in Section 3. In Section 4, the experimental results are presented, including the performance of activity recognition and the amount of fluid intake estimation. The effect and potentiality of the proposed system are discussed in Section 5. Finally, the conclusion is presented in Section 6.

## 2. Related Work

Many studies have proposed approaches to monitor fluid intake by wearable inertial sensors. There are two main topics for fluid intake monitoring, including drinking activity recognition and fluid intake amount estimation. The related literature will be described in the following subsections.

### 2.1. Drinking Activity Recognition

There are two categories of drinking activity recognition, including ambient-based and wearable-based. Ambient-based drinking activity recognition utilizes sensors installed in environments or objects to detect drinking activities. Iosifidis et al. [15] used a camera placed in front of a person to capture a meal intake process. Then, linear discriminant analysis (LDA) was applied to classify the eating and drinking activities. Tham et al. [16] obtained depth information from a camera and recognized drinking activities by dynamic time warping (DTW). However, cameras can only monitor activities in a confined space and may have privacy issues when installed in environments. Jayatilake et al. [17] proposed a real-time drinking recognition system with passive radio frequency identification (RFID) tags attached to fluid containers and a RFID antenna mounted near the ceiling. The classification using non-linear support vector machine (SVM) achieved 87% F- measure. Liu et al. [15] designed a 3D-printed smart cup attached with an accelerometer to detect drinking events. The best performance was 89.9% F-measure by *k*-nearest neighbors (*k*-NN) model. Despite the high F-measure, with these approaches there exist limitations in usage of certain containers and fixed users that may cause inconvenience to users. 

Alternatively, wearable-based drinking activity recognition utilizes acoustic and inertial sensors worn on the human body to detect drinking activities. Bi et al. [19] recognized water swallowing events and other food chewing events by a neck-worn microphone. Hidden Markov models were utilized to identify chewing or swallowing events. Rahman et al. [18] developed a neckpiece equipped with a microphone and applied LDA to classify drinking activities and other daily activities. The sensitivity and precision of classification is 72% and 57%, respectively. Gomes et al. [25] recognized drinking and eating by a wrist-worn sensor. The best performance of classification was 86% accuracy using random forest (RF). Chun et al. [26] employed an adaptive segmentation technique and various machine-learning-based classifiers to detect fluid intake. The system obtained the best results of 90.3% precision and 91.0% sensitivity by using the RF model. Wearable-based drinking activity recognition demonstrates good performance by wrist-worn inertial sensors. In addition, wrist-worn inertial sensors can not only recognize motion information, but also estimate the intake amount. 

### 2.2. Fluid Intake Amount Estimation

Fluid intake amount estimation is important for fluid intake monitoring. There are two types of approaches for intake amount estimation. Firstly, a container embedded with inertial sensors is a widely used approach to estimate fluid intake amount. Zimmermann et al. [27] designed a smart cup holder embedded with an accelerometer and a gyroscope to measure the movement and a force sensor to detect the consumed amount. Griffith et al. [28] computed drinking volume by a bottle attached with a tri-axial accelerometer and gyroscope. SVM regression models estimated intake volume and achieved 52.4% mean absolute percentage error (MAPE). Secondly, few papers utilize wearable inertial sensors to estimate intake amount. Hamatani et al. [24] used inertial sensors of smartwatches and a linear regression model to estimate the intake amount. The study was conducted experiments in the laboratory and in real life, unobtrusively, and applied smart bottles to provide the ground truth of drinking amount. The results presented 31.8% and 34.6% MAPE in the laboratory and in real life, respectively.

## 3. Materials and Methods

**Figure 1:**
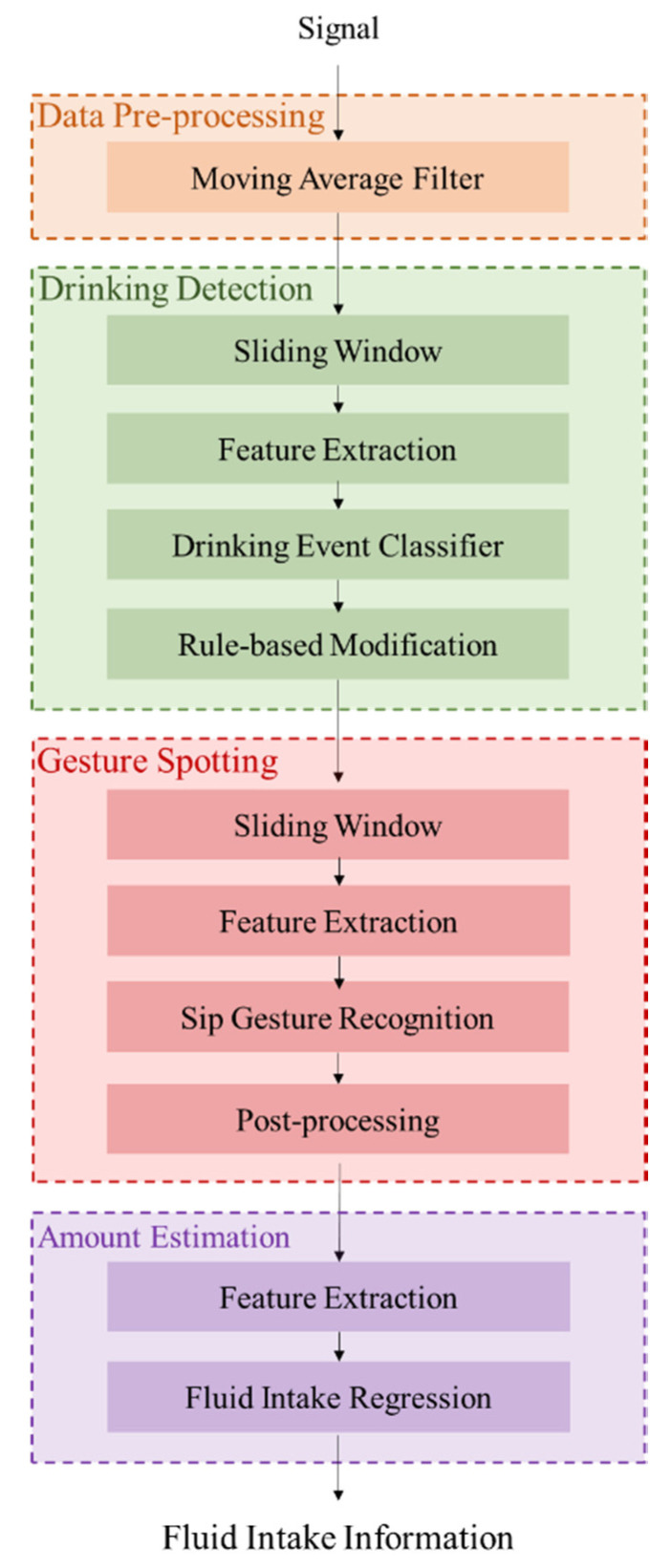
The system architecture of the proposed fluid intake monitoring system.

To monitor fluid intake automatically, drinking activity detection and fluid intake estimation are important topics. The previous studies only focus on one topic independently. However, the comprehensive analysis should be considered. This work proposes a hierarchical fluid intake monitoring system using a wearable inertial sensor to acquire detailed gesture information and apply elaborate features for fluid intake estimation. The system architecture of the proposed fluid intake monitoring system is shown in Figure 1. The fluid intake monitoring system consists of four functional components, including data pre-processing, drinking detection, gesture spotting and amount estimation. Firstly, the movement signal was collected by a wearable inertial sensor worn on the wrist. A moving average filter was applied to reduce the noise effects. Secondly, drinking detection utilizes a machine-learning-based classifier and rule-based modification to detect drinking activities. Furthermore, gesture spotting recognizes sip gesture from detected drinking activities. Finally, the signal of sip gestures was utilized to estimate amount of fluid intake based on the regression model.

### 3.1. Data Acquisition and Experimental Protocols

**Figure 2:**
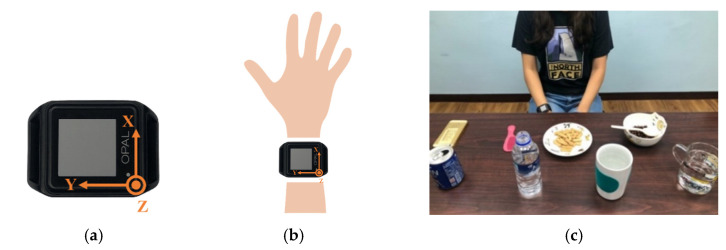
The orientation and position of a sensor in the experiment. (**a**) The orientation of an OPAL sensor. (**b**) The sensor worn on the right wrist. (**c**) The initial position of the sensor.

An OPAL sensor, published by APDM, Portland, USA, was utilized to collect the motion data. A tri-axial accelerometer, gyroscope and magnetometer were embedded in the OPAL sensor. However, considering the influence of magnetic disturbances on the magnetometer, only the accelerometer (range ±16 G) and gyroscope (range ±2000 degree/s) were utilized for fluid intake activity monitoring system. The sampling rate was 128 Hz and the orientation of OPAL sensor is shown in Figure 2a. Two smartphones (iPhone 6, published by Apple, Cupertino, CA, USA) synchronized with the OPAL sensor were applied to record videos to provide reference data during the entire experiment. With the information from cameras including starting and ending time of each activity, the researcher can label the ground truth manually to evaluate the performance of fluid intake activity monitoring system. The smartphones were placed at the front and lateral side of the subjects. The sampling rate of the camera was 30 Hz.

Twenty participants were recruited in this study (9 males, 11 females, 24.6 ± 3.6 years, height = 168.4 ± 9.4 cm, weight = 63.6 ± 13.9 kg). The participant performed trials sitting on a chair with a single inertial sensor worn on the right wrist, as shown in Figure 2b,c. Each trial was a combination of activities, including answering a phone call (A), combing hair (C), eating with hands (H), eating with a spoon (S) and fluid intake (FI). These activities were similar in motions towards head or mouth. Each activity would be accomplished with specific objects put on the table. Therefore, participants had to take the object on the table, move it towards the body, perform the specific activities and put the object back to the table.

**Figure 3:**
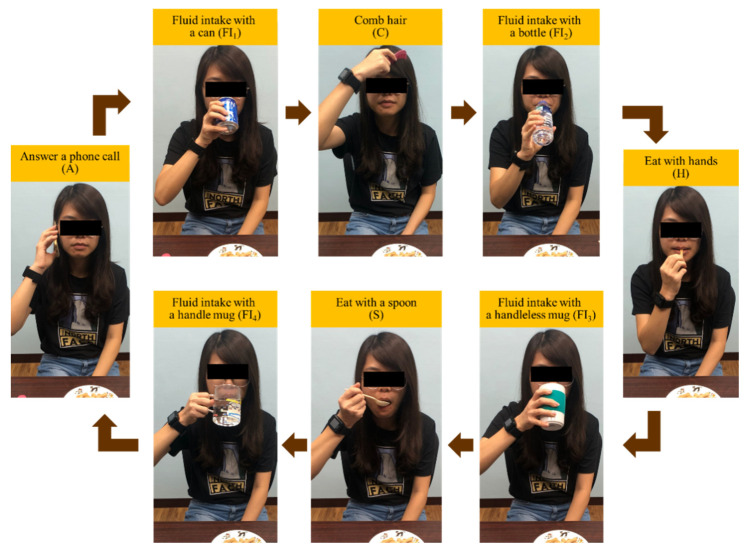
The performed activity sequence in the experiments: answer a phone call (A) → fluid intake with a can (FI_1_) → comb hair (C) → fluid intake with a bottle (FI_2_) → eat with hands (H) → fluid intake with a handleless mug (FI_3_) → eat with a spoon (S) → fluid intake with a handled mug (FI_4_) → answer a phone call (A).

**Figure 4:**
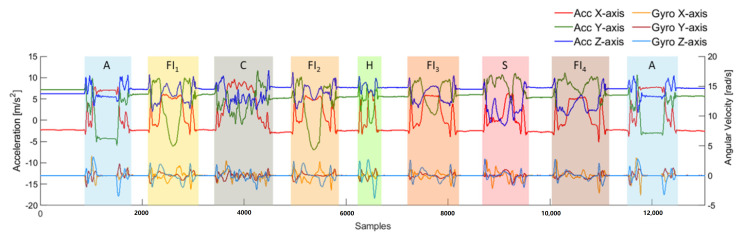
An example of the collected signal through the accelerometer (Acc) and gyroscope (Gyro) by a subject. The colored bar is the ground truth of activities, including answering a phone call (A), fluid intake with a can (FI_1_), combing hair (C), fluid intake with a bottle (FI_2_), eating with hands (H), fluid intake with a handleless mug (FI_3_), eating with a spoon (S), fluid intake with a handled mug (FI_4_).

To evaluate the influence of drinking with different types of container, four FI activities were involved in one trial and each FI activity was executed with a different container, including a can (FI_1_), a plastic bottle (FI_2_), a handleless mug (FI_3_) and a handled mug (FI_4_). As shown in Figure 3, the activities were performed in the following sequences: A → FI_1_ → C → FI_2_ → H → FI_3_ → S → FI_4_ → A. Figure 4 shows an example of the trial performed by a subject. The example demonstrates the differences between containers, especially fluid intake with handleless mugs and with handled mugs. To obtain true amounts of fluid intake activities, a kitchen scale was used to measure the weight of each container before the trial was started and after the trial was finished. 

A participant would perform a trial five times. Therefore, in total 900 activities were collected (9 activities × 5 trials × 20 participants). The initial filling levels were 100% (240 g/240 g), 100% (330 g/330 g), 87.5% (280 g/320 g) and 70% (300 g/430 g) for cans, bottles, handleless mugs and handled mugs, respectively. At the first trial, subjects drank the water from the initial filling level. Then, at the second and third trial, subjects might drink from different filling levels depending on the previous intake volumes. The water in containers was refilled before the fourth trial. Hence, subjects would drink the water from the initial filling level at the fourth trial. Finally, the last trial was performed with different filling levels for each subject. There were two identical filling levels and three diverse filling levels performed by subjects.

### 3.2. Data Pre-Processing

A wrist-worn wearable sensor embedded with tri-axial accelerometer and gyroscope was applied to acquire sensing data of fluid intake activities. However, the sensing data from the accelerometer and gyroscope of the wearable sensor were affected by the muscle vibration and disturbance of the environment. These confusing data may lead to the difficulties of fluid intake recognition. A moving average filter was utilized to reduce the noise with 16 data samples.

### 3.3. Drinking Detection

Drinking detection classifies drinking activities among activities of daily living. Drinking activities were detected by four steps: sliding window, feature extraction, drinking event classifier and rule-based modification. Firstly, the smoothed data were segmented by the sliding window technique. The sliding window technique is widely used to partition the continuous sensing data into segments. However, different window size and overlap percentage may affect the performance of the activity recognition. To determine a proper window size and overlap percentage, various window sizes ranging from 64 to 256 data samples with step size of 32 samples and various overlap percentage including 25%, 50% and 75% were adopted for drinking detection. 

Secondly, eight types of statistical features were extracted, including mean, standard deviation, variance, maximum, minimum, range, kurtosis and skewness. These eight types of features were extracted from each axis of the accelerometer and gyroscope, Euclidean norm of tri-axial acceleration, Euclidean norm of tri-axial gyroscope, and Euclidean norm of acceleration in the horizontal, coronal, and sagittal plane, as calculated by Equations (1)–(5), where $a_{x}$ is the acceleration of *x*-axis, $a_{y}$ is the acceleration of *y*-axis, $a_{z}$ is the acceleration of *z*-axis, $\omega_{x}$ is the angular velocity of *x*-axis, $\omega_{y}$ is the angular velocity of *y*-axis, and $\omega_{z}$ is the angular velocity of *z*-axis. There were, in total, 88 features (8 types × 11 axes) extracted from the segmented data, as shown in Table 1.

(1) $$a_{norm}=\;\sqrt{a_{x}^{2}+a_{y}^{2}+a_{z}^{2}}$$

(2) $$\omega_{norm}=\;\sqrt{\omega_{x}^{2}+\omega_{y}^{2}+\omega_{z}^{2}}$$

(3) $$a_{norm,xy}=\;\sqrt{a_{x}^{2}+a_{y}^{2}}$$

(4) $$a_{norm,xz}=\;\sqrt{a_{x}^{2}+a_{z}^{2}}$$

(5) $$a_{norm,yz}=\;\sqrt{a_{y}^{2}+a_{z}^{2}}$$

Thirdly, drinking event classifier utilizes machine learning models to identify drinking activities and other activities. The starting and ending time of drinking activities during consecutive activities of daily living can be detected in this step. Six machine learning models were adopted, including Adaptive Boosting (AdaBoost), decision tree (DT), random forest (RF), Naïve Bayes (NB), *k*-nearest neighbor (*k*-NN) and support vector machine (SVM). The brief introduction of these machine learning models is as follows:

1. Adaptive Boosting (AdaBoost)
  AdaBoost is an ensemble learning technique. By multiple weak models and weights of training samples, AdaBoost can construct a strong classifier. At each iteration of the training progress, a higher weighting is assigned to the misclassified data of the weak classifier and a lower weighting is allocated to the correctly classified data. The data with weights are utilized to train the next weak classifier. These weak classifiers are combined and the final class is decided by a weighted sum of the weak classifiers. In this study, the strong classifier is an ensemble of weak decision trees using classification and regression trees (CARTs).
2. Decision Tree (DT)
  DT is a classical model to classify the data. The tree-like model generates decision nodes and leaf nodes based on rules and thresholds. The data can be classified by following the nodes. In this work, CARTs based on impurity are utilized to classify the data.
3. Random Forest (RF)
  RF combines multiple decision trees based on the bagging technique to solve the overfitting problem of decision tree. Firstly, the model randomly selects a subset of training data. Next, a decision tree is trained by the subset. Finally, the previous two steps repeat iteratively to generate multiple decision trees. The final class are decided by a majority vote. The decision trees in RF are implemented based on CARTs with minimum leaf size of 1 and minimum parent size of 2.
4. Naïve Bayes (NB)
  NB models classify data based on Bayes’ theorem. The probabilistic model states the independence between extracted features. The distribution of the features must be assumed. Then, the final class can be predicted by maximum probability of the class. In this work, different distributions (e.g., multinomial distribution, multivariate multinomial distribution and normal distribution) are tested, and the NB model with normal distribution reaches the best performance.
5. *K*-nearest Neighbor (*k*-NN)
  *K*-nearest neighbor model is a simple method for classification. The *k*-NN model calculates the distance between data and decides the class by the majority vote of the closest *k* training instances. In this work, a range of *k* from 1 to 15 is explored to find the best performance using *k*-NN. The results show that the best performance of drinking detection is achieved by *k* = 3.
6. Support Vector Machine (SVM)
  An SVM model is one of the widely used supervised machine learning models for classification. The SVM model calculates the separating hyperplane that has the maximum distance between two classes of data. The classification can be determined by the hyperplane. In this work, a liner kernel function is applied to the SVM model.

**Figure 5:**
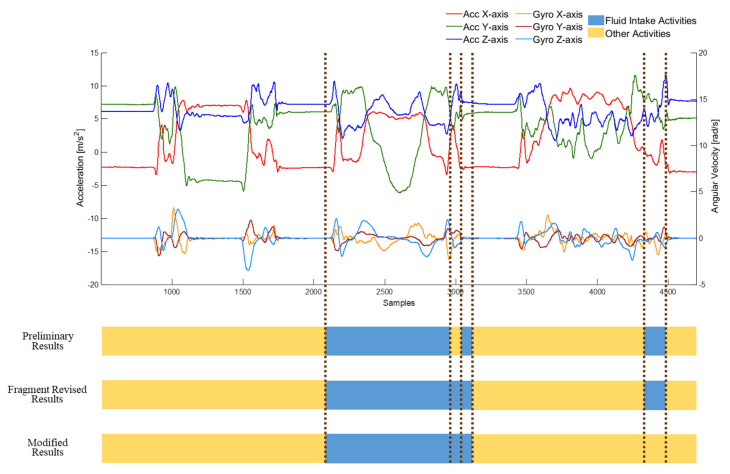
An example of detection results after rule-based modification, including fragment revision and duration threshold.

**Figure 6:**
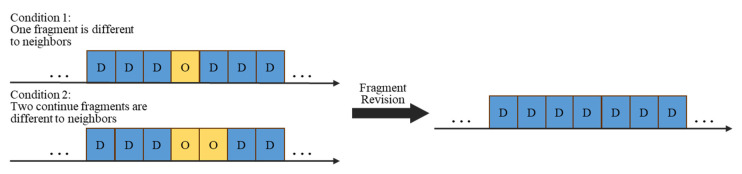
An example of fragment revision to revise one or two continued fragments that are different to neighbors. D represents drinking activities and O represents other activities.

Finally, rule-based modification was implemented to modify the misclassification of the drinking event classifier. An example of modification is shown in Figure 5. There were two stages of modification. In the beginning, fragment revision deals with the segments with inconsistent predicted results to the neighbors. Because of the misclassification of the drinking event classifier, there may be fragments of other activities identified in a complete drinking event. Therefore, fragment revision was applied to deal with these misclassified fragments. If the predicted results of a fragment or two continue fragments were different to the previous and the subsequent fragment, and the results of the previous and subsequent ones were identical, the fragment was regarded as the misclassified fragment. Then, the misclassified fragment should be revised to the classified result of the previous fragment, as shown in Figure 6. After the fragment revision, a duration threshold was applied to identify the drinking activities. The duration of fluid intake activity should be longer than 2 s for participants to finish the complete fluid intake. Therefore, in rule-based modification, the predicted results were modified by identifying the lasting time of detected drinking activities. In other words, if the duration of the detected fluid intake activity was less than 2 s, the detected result was considered as the misclassified activity and modified to the other activity. 

### 3.4. Gesture Spotting

Gesture spotting is implemented to acquire the detailed gesture information of drinking activities for amount estimation. A drinking activity can be divided into five subtasks including fetching the container, lifting the container to mouth, having a sip, dropping the container back to the initial position and releasing the container. Gesture spotting aims to recognize the sip gestures from these subtasks. The motion and duration of sip periods can provide important information for fluid intake estimation.

There are four steps in gesture spotting: sliding window, feature extraction, sip gesture recognition and post-processing. Firstly, a sliding window technique segments the signal of the drinking activity. To find a proper window size and overlap percentage, various window sizes and overlapping are adopted. The adopted window sizes are 4, 8, 16, 32 and 64 data samples and the overlap percentages are 25%, 50% and 75%.

Secondly, feature extraction is utilized to obtain the key features for gesture spotting. The detailed information of features is shown in Table 1. As features of drinking detection, eight types of statistical features are extracted from the segmented data, which are mean, standard deviation, variance, maximum, minimum, range, kurtosis and skewness. These features are extracted from eleven axes, including each axis of the accelerometer and gyroscope, Euclidean norm of tri-axial acceleration, Euclidean norm of tri-axial gyroscope, and Euclidean norm of acceleration in the horizontal, coronal, and sagittal plane. Therefore, there are, in total, 88 features (8 types × 11 axes) extracted from the segmented data for gesture spotting. 

Furthermore, machine learning models recognize gestures of the drinking activity, including *Fetch* (fetch the container from the table), *Lift* (lift the container towards the mouth), *Sip* (have a sip), *Drop* (put the container back to the table), and *Release* (return the hand back). Six machine learning models were implemented, including AdaBoost, DT, RF, NB, *k*-NN and linear SVM. The brief introduction of these models is presented in Section 3.3. By exploring *k* parameter from 1 to 15, the best performance of *k*-NN is achieved using *k* = 11. 

Finally, the predicted fragment that is not consistent with the previous and the subsequent one was modified in post-processing. As the fragment revision of drinking detection shown in Figure 6, the results of one fragment or two continue fragments that are not consistent to that of the previous and subsequent one are modified to the result of the previous fragment. 

### 3.5. Amount Estimation

Fluid intake amount can be estimated by the sip gesture information from gesture spotting. In amount estimation, the whole signals of a sip gesture are employed to evaluate the drinking amount. Nine types of features were extracted for amount estimation, including mean, standard deviation, variance, maximum, minimum, range, kurtosis, skewness and duration. As shown in Table 2, the previous eight types of features were calculated from eleven axes as drinking detection and gesture spotting, and the duration was the length of time during a sip. Therefore, there were 89 features (8 types × 11 axes + 1 type) extracted from the recognized sip data for amount estimation. 

After feature extraction, various container-dependent amount estimation models were implemented for each container, including cans, bottles, handleless mugs and handled mugs. Moreover, five regression models were executed and tested to estimate the fluid intake amount (e.g., linear regression, Gaussian kernel regression, and SVM regression with a linear kernel (SVM-linear), a polynomial kernel of degree 3 (SVM-Poly) and a Radial Basis Function kernel (SVM-RBF). 

### 3.6. Performance Evaluation

Leave-one-subject-out (LOSO) cross validation approach was employed to validate the performance of the proposed fluid intake monitoring system. Moreover, various metrics were applied to evaluate the performance of drinking detection, gesture spotting and amount estimation of the proposed hierarchical approach.

For drinking detection, four metrics were utilized to evaluate the performance, including sensitivity, precision, specificity and accuracy. These metrics were calculated by Equations (6)–(9). The drinking event classifier may classify the sensing data into positive and negative, which means drinking activities and other activities, respectively. Therefore, there were four situations in drinking detection, including true positive (TP), false positive (FP), true negative (TN) and false negative (FN), which represented that the labelled drinking activity was detected as drinking, the other labelled activity was detected as drinking activity, the other labelled activity was detected as other activity and the labelled drinking activity was detected as other activity.

(6) $$Sensitivity=\frac{TP}{TP+FN}$$

(7) $$Precision=\frac{TP}{TP+FP}$$

(8) $$Specificity=\frac{TN}{TN+FP}$$

(9) $$Accuracy=\frac{TP+TN}{TP+FP+TN+FN}$$

Two metrics, sensitivity and precision, were used to assess the performance of gesture spotting. Since there were five gestures recognized in the gesture spotting, metrics of sensitivity and precision were calculated for each gesture. Therefore, there may be 10 metrics (5 gestures × 2 metrics) demonstrated, including *Fetch* sensitivity and precision, *Lift* sensitivity and precision, *Sip* sensitivity and precision, *Drop* sensitivity and precision, and *Release* sensitivity and precision. Moreover, the overall sensitivity and precision were presented to choose the best recognition model for amount estimation.

To evaluate the performance of fluid intake estimation, mean percentage error (MPE) and mean absolute percentage error (MAPE) were employed. MPE is an average of percentage errors for each intake. The calculation of MPE is shown in Equation (10), where n is the length of data, $e_{i}\;$is the estimated amount of *i*th intake, and $a_{i}$ is the actual amount of *i*th intake. The positive and negative errors involving in MPE may offset each other. On the contrary, MAPE provides a rigorous evaluation on the performance. As shown in Equation (11), absolute errors are calculated for each intake. MAPE indicates the difference between the estimated value and actual value.

(10) $$\mathrm{MPE}=\frac{1}{n}\overset{n}{\underset{i=1}{\sum }}\frac{a_{i}-e_{i}}{a_{i}}\times 100\%$$

(11) $$\mathrm{MAPE}=\frac{1}{n}\overset{n}{\underset{i=1}{\sum }}\left|\frac{a_{i}-e_{i}}{a_{i}}\right|\times 100\%$$

## 4. Results

In this work, a fluid intake monitoring system is proposed to estimate fluid intake amount by a hierarchical approach. The experimental results of the proposed system are divided into two parts. Results of the activity recognition, which includes drinking detection and gesture spotting, are demonstrated in the beginning. Experimental results of the amount estimation through the hierarchical approach are then presented.

### 4.1. Drinking Detection and Gesture Spotting

In drinking detection, six machine learning models and various combinations of window sizes and overlaps are applied to detect drinking activities. The best performance of drinking detection using different machine learning models is shown in Table 3. The experimental results show that window sizes with 50% overlapping demonstrate better performance than that with other overlap percentage for each model, except the k-NN model. However, the k-NN model with 128 window sizes and 50% overlapping can reach 93.66% accuracy, which is close to the accuracy of the k-NN model using 25% overlapping. 

**Figure 7:**
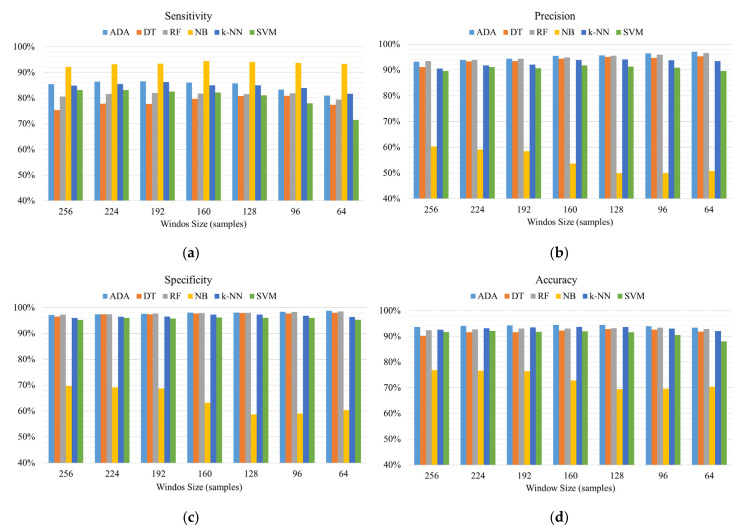
The detection performance of (**a**) sensitivity, (**b**) precision, (**c**) specificity and (**d**) accuracy of using different machine learning models and sliding window techniques with 50% overlapping.

Therefore, we only demonstrate the performance of models using the overlap of 50%. As shown in Figure 7, AdaBoost (ADA) models outperform other machine learning models with the best accuracy of 94.42%. Although NB models achieve the best sensitivity, the accuracy of NB models underperforms that of other models. The results demonstrate that AdaBoost (ADA), with a window size of 160 samples and an overlap of 50%, achieves the best performance with 94.42% accuracy, 86.06% sensitivity, 95.50% precision and 98.08% specificity. 

**Figure 8:**
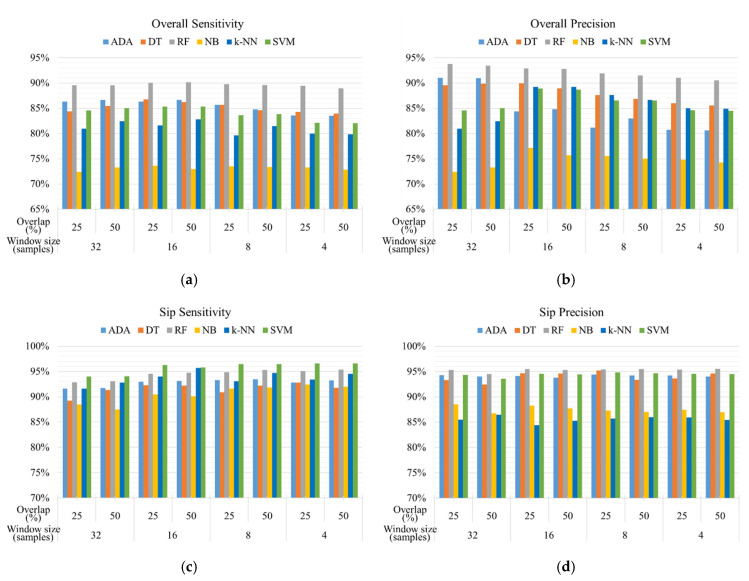
The recognition performance of (**a**) overall sensitivity and (**b**) overall precision of all gestures, and (**c**) sensitivity and (**d**) precision of sip gesture using different machine learning models and sliding window techniques.

Gesture spotting aims to recognize sip gesture from the drinking activities and provide the critical sip information for amount estimation. Six machine learning models with various window sizes and overlaps are utilized to recognize *Fetch* (fetch the container from the table), *Lift* (lift the container towards the mouth), *Sip* (have a sip), *Drop* (put the container back to the table), and *Release* (return the hand back) gestures. As shown in Figure 8, the overall performance of RF models outperforms that of other models in overall sensitivity and precision. However, the sensitivity of sip gesture recognition using RF models is lower than that using SVM models. Table 4 demonstrates the best performance of gesture spotting using different machine learning models. The RF model with a window size of 16 samples and 50% overlapping achieves the best overall sensitivity (90.17%) and overall precision (92.80%). Despite the high sensitivity of sip gesture by the *k*-NN and SVM model, the sensitivity between different gestures ranges from 71.58% to 96.28%. However, gesture spotting using the RF model can achieve approximately 90% sensitivity for each gesture.

### 4.2. Amount Estimation

This work designed container-dependent amount estimation models for each container to enhance the reliability of the proposed fluid intake monitoring system. Five regression models are applied to estimate the intake amount of each container-dependent estimation model. In addition, a container-independent amount, the estimation model is implemented to compare the performance of amount estimation with container-dependent models. 

Table 5 shows the performance of amount estimation using different models. The results demonstrate that the container-independent model using SVM regression with linear kernel function can achieve better performance with 12.68% MPE and 40.06% MAPE than the typical linear regression model (28.96% MPE and 49.53% MAPE). In addition, most container-dependent amount estimation models present better performance than the container-independent model. The best MAPE improvement of container-dependent models is 10.53% for cans using SVM-linear, 7.01% for bottles using SVM-RBF, 6.61% for handleless mugs using SVM-Poly and 5.01% for handled mugs using SVM-Poly, compared to container-independent models. The experimental results reveal that the container information can provide critical information for amount estimation. For container-dependent estimation, the performance of SVM-linear outperforms that of other regression models, except using handled mugs. However, the difference between MAPE of different regression models using handled mugs is within 6%. The average MAPE of four container-dependent estimation models using SVM-linear is 37.36%, which is the lowest MAPE among other regression models. 

The proposed fluid intake monitoring system utilizes a hierarchical approach to acquire drinking activities, sip gestures and amount estimation. To evaluate the estimation performance, two situations are designed: (1) amount estimation by information of drinking activities only, and (2) amount estimation by information of sip gesture. In situation (1), a drinking activity is directly employed to estimate fluid intake amount. According to Table 3, the AdaBoost model, that achieves the best performance of drinking detection, is firstly utilized to detect drinking activities from other activities. Next, the detected results of the AdaBoost model are directly applied to the SVM regression model with linear kernel function for amount estimation. The extracted features for amount estimation are shown in Table 2. The duration of drinking activities is extracted for substituting sip duration. For situation (2), the hierarchical approach is applied to estimate intake amount. First, the AdaBoost model with a window size of 160 samples and an overlap of 50% is employed to detect drinking activities. Next, the RF model with a window size of 16 samples and an overlap of 50% recognizes the sip gestures from drinking activities. Finally, the features extracted from sip gestures are utilized to estimate amount by an SVM regression model with linear kernel function.

The results of two situations are shown in Table 6. With sip gesture information, the container-independent amount estimation can be reduced to −12.34% MPE and 40.11% MAPE, which is an improvement of 22.51% and 25.75% compared to that with drinking activity only. Moreover, container-dependent amount estimation model in situation (2) can achieve lower MAPE than that in situation (1). The results demonstrate that the sip gesture information is critical to fluid intake estimation. In situation (2), most container-dependent amount estimation models demonstrate better MAPE than container-independent model. However, the amount estimation of drinking with cans presents a large MAPE among the four containers. 

## 5. Discussion

The main objective of this work is to propose a fluid intake monitoring system with a wearable inertial sensor for detecting fluid intake activities, recognizing sip gestures and estimating fluid intake amount. Previous studies have proposed various approaches using wrist-worn inertial sensors for detecting fluid intake activities and achieved good detection performance [11,24,25]. However, few works focus on estimation of fluid intake amount. This work applies a hierarchical approach to the fluid intake monitoring system to obtain information of drinking activities, sip gestures and intake amounts. Furthermore, container-dependent and -independent amount estimation models are implemented to estimation drinking amount. The results reveal that the sip information can improve approximately 20% MAPE of amount estimation. Moreover, the container-dependent amount estimation models can enhance the reliability of amount estimation. However, the misclassification of drinking activity recognition may affect the performance of amount estimation.

The experimental results demonstrate that drinking detection using the AdaBoost model can achieve 86.06% sensitivity and 94.42% accuracy and gesture spotting using the RF model can get an overall sensitivity of 90.17% and overall precision of 92.80%. This work has the similar performance to the previous studies [11,24]. However, the misclassification of drinking detection affects the performance of amount estimation negatively. First, most misclassification occurs at the beginning and ending of the fluid intake activity. Second, other activities may be misclassified as fluid intake activities. Such misclassification may accumulate the total errors of the amount estimation. Particularly, eating activities with spoons are easily misclassified as fluid intake activities because they have similar motion patterns. Last, the detection of drinking activities with cans have the worst performance than that with other containers as several subjects have the unique drinking movement patterns. The significant differences cause the errors in drinking activities detection and intake amount estimation with cans. To enhance the detection performance, neural network approaches (e.g., convolutional neural networks [29] and recurrent neural networks [30]) would be implemented in future work. 

To enhance the amount estimation, this work applies and tests machine-learning-based regression models and container-dependent amount estimation models to the proposed system. The performance of amount estimation using the linear SVM regression model improves 10% MAPE compared to the linear regression model. The reason is that SVM regression model can reduce the negative offset of outlier data by constructing the hyperplane. In contrast, the linear regression model is easily affected by the bias. The container-dependent amount estimation model can improve the MAPE, ranging from 5.01% to 10.53%, compared to the container-independent models. Moreover, amount estimation using the sip gesture information has an improvement of 25.75% MAPE, while compared to using the drinking activities. The results reveal that the sip and container information is critical to the fluid intake monitoring system.

However, the fluid intake amount estimation is still challengeable. Firstly, the individual difference in drinking amount causes the variability of motion signals. The motion patterns might be different even though the subjects consume the same amount of water. Secondly, there may be differences between the motion signals of large intake amount and small intake amount. It is not suitable to use identical models to gauge the intake amount of large and small sip sizes. Finally, the diverse filling levels influence the estimation performance. The inclination of the head and the wrist may be different while drinking from different filling levels. The subject needs to lean at a larger angle to drink water at a small filling level than that at a large filling level. To enhance the performance of fluid intake monitoring system, this work will include more factors that may influence intake amount, such as sip sizes and fill levels. 

There are some limitations in this work. Firstly, more factors associated with drinking, such as sip size and filling level, should be included into amount models. Second, the feasibility of the proposed systems in different groups will be explored, including old people and patients with chronic diseases. Additionally, the advanced machine learning models (e.g., deep learning approaches) will be applied to tackle the variability of drinking behavior affected by ages, diseases, and gender. Finally, the current experiments are conducted in a laboratory environment. Fluid intake monitoring during free-living needs to be evaluated in the future.

## 6. Conclusions

Fluid intake management can provide fluid intake information for people to realize and manage their fluid intake behaviors. However, few studies focus on the estimation of fluid intake amount. This work presents a fluid intake monitoring system with a wearable inertial sensor using hierarchical machine learning models to detect fluid intake activities, recognizing sip gestures and estimating the fluid intake amount. Through the drinking detection and gesture spotting, the critical sip information can be recognized. The sip information is further utilized to estimate the amount of fluid intake. In addition, the container-dependent amount estimation model is proposed to enhance the reliability of the amount estimation. The proposed fluid intake monitoring system could achieve 94.42% accuracy, 90.17% sensitivity, and 40.11% MAPE for drinking detection, gesture spotting and amount estimation, respectively. Particularly, MAPE of amount estimation is improved approximately 10% compared to the typical approaches. The results have demonstrated the feasibility and the effectiveness of the proposed fluid intake monitoring system.

## Figures and Tables

**Table 1:** The list of features extracted for drinking detection and gesture spotting.

Features	Description
$f_{1}$–$f_{11}$	Mean of $a_{x}$, $a_{y}$, $a_{z}$, $\omega_{x}$, $\omega_{y}$, $\omega_{z}$, $a_{norm}$, $\omega_{norm}$, $a_{norm,xy}$, $a_{norm,yz}$, $a_{norm,xz}$
$f_{12}$–$f_{22}$	Standard deviation of $a_{x}$, $a_{y}$, $a_{z}$, $\omega_{x}$, $\omega_{y}$, $\omega_{z}$, $a_{norm}$, $\omega_{norm}$, $a_{norm,xy}$, $a_{norm,yz}$, $a_{norm,xz}$
$f_{23}$–$f_{33}$	Variance of $a_{x}$, $a_{y}$, $a_{z}$, $\omega_{x}$, $\omega_{y}$, $\omega_{z}$, $a_{norm}$, $\omega_{norm}$, $a_{norm,xy}$, $a_{norm,yz}$, $a_{norm,xz}$
$f_{34}$–$f_{44}$	Maximum of $a_{x}$, $a_{y}$, $a_{z}$, $\omega_{x}$, $\omega_{y}$, $\omega_{z}$, $a_{norm}$, $\omega_{norm}$, $a_{norm,xy}$, $a_{norm,yz}$, $a_{norm,xz}$
$f_{45}$–$f_{55}$	Minimum of $a_{x}$, $a_{y}$, $a_{z}$, $\omega_{x}$, $\omega_{y}$, $\omega_{z}$, $a_{norm}$, $\omega_{norm}$, $a_{norm,xy}$, $a_{norm,yz}$, $a_{norm,xz}$
$f_{56}$–$f_{66}$	Range of $a_{x}$, $a_{y}$, $a_{z}$, $\omega_{x}$, $\omega_{y}$, $\omega_{z}$, $a_{norm}$, $\omega_{norm}$, $a_{norm,xy}$, $a_{norm,yz}$, $a_{norm,xz}$
$f_{67}$–$f_{77}$	Kurtosis of$\;a_{x}$, $a_{y}$, $a_{z}$, $\omega_{x}$, $\omega_{y}$, $\omega_{z}$, $a_{norm}$, $\omega_{norm}$, $a_{norm,xy}$, $a_{norm,yz}$, $a_{norm,xz}$
$f_{78}$–$f_{88}$	Skewness of $a_{x}$, $a_{y}$, $a_{z}$, $\omega_{x}$, $\omega_{y}$, $\omega_{z}$, $a_{norm}$, $\omega_{norm}$, $a_{norm,xy}$, $a_{norm,yz}$, $a_{norm,xz}$

**Table 2:** The list of features extracted for amount estimation.

Features	Description
$f_{1}$–$f_{11}$	Mean of $a_{x}$, $a_{y}$, $a_{z}$, $\omega_{x}$, $\omega_{y}$, $\omega_{z}$, $a_{norm}$, $\omega_{norm}$, $a_{norm,xy}$, $a_{norm,yz}$, $a_{norm,xz}$
$f_{12}$–$f_{22}$	Standard deviation of $a_{x}$, $a_{y}$, $a_{z}$, $\omega_{x}$, $\omega_{y}$, $\omega_{z}$, $a_{norm}$, $\omega_{norm}$, $a_{norm,xy}$, $a_{norm,yz}$, $a_{norm,xz}$
$f_{23}$–$f_{33}$	Variance of $a_{x}$, $a_{y}$, $a_{z}$, $\omega_{x}$, $\omega_{y}$, $\omega_{z}$, $a_{norm}$, $\omega_{norm}$, $a_{norm,xy}$, $a_{norm,yz}$, $a_{norm,xz}$
$f_{34}$–$f_{44}$	Maximum of $a_{x}$, $a_{y}$, $a_{z}$, $\omega_{x}$, $\omega_{y}$, $\omega_{z}$, $a_{norm}$, $\omega_{norm}$, $a_{norm,xy}$, $a_{norm,yz}$, $a_{norm,xz}$
$f_{45}$–$f_{55}$	Minimum of $a_{x}$, $a_{y}$, $a_{z}$, $\omega_{x}$, $\omega_{y}$, $\omega_{z}$, $a_{norm}$, $\omega_{norm}$, $a_{norm,xy}$, $a_{norm,yz}$, $a_{norm,xz}$
$f_{56}$–$f_{66}$	Range of $a_{x}$, $a_{y}$, $a_{z}$, $\omega_{x}$, $\omega_{y}$, $\omega_{z}$, $a_{norm}$, $\omega_{norm}$, $a_{norm,xy}$, $a_{norm,yz}$, $a_{norm,xz}$
$f_{67}$–$f_{77}$	Kurtosis of$\;a_{x}$, $a_{y}$, $a_{z}$, $\omega_{x}$, $\omega_{y}$, $\omega_{z}$, $a_{norm}$, $\omega_{norm}$, $a_{norm,xy}$, $a_{norm,yz}$, $a_{norm,xz}$
$f_{78}$–$f_{88}$	Skewness of $a_{x}$, $a_{y}$, $a_{z}$, $\omega_{x}$, $\omega_{y}$, $\omega_{z}$, $a_{norm}$, $\omega_{norm}$, $a_{norm,xy}$, $a_{norm,yz}$, $a_{norm,xz}$
$f_{89}$	Duration of the sip gesture

**Table 3:** The best performance of drinking detection using different machine learning models.

Machine Learning Model	Window Size (Samples)	Overlap (%)	Sensitivity (%)	Precision (%)	Specificity (%)	Accuracy (%)
ADA	160	50	86.06	95.50	98.08	94.42
DT	128	50	80.83	95.05	97.91	92.79
RF	96	50	81.87	95.96	98.29	93.34
NB	256	50	92.20	60.28	69.75	76.76
*k*-NN	128	25	84.87	94.29	97.45	93.68
SVM	224	50	83.17	91.07	96.02	92.14

ADA: AdaBoost; DT: decision tree; RF: random forest; NB: Naïve Bayes; *k*-NN: *k*-nearest neighbors; SVM: support vector machine.

**Table 4:** The best performance of gesture spotting using different machine learning models.

Machine Learning Model	Window Size (Samples)	Overlap (%)	Metric	Gesture					Overall
				Fetch	Lift	Sip	Drop	Release	
ADA	16	50	Sensitivity (%)	83.26	89.74	93.10	90.62	76.58	86.66
			Precision (%)	85.64	91.55	93.83	89.88	85.26	89.23
DT	16	25	Sensitivity (%)	89.72	84.08	92.24	86.98	80.79	86.76
			Precision (%)	84.03	91.92	94.66	91.61	87.53	89.95
RF	16	50	Sensitivity (%)	89.35	88.70	94.75	90.61	87.44	90.17
			Precision (%)	91.63	95.27	95.35	93.98	87.80	92.80
NB	16	25	Sensitivity (%)	57.35	77.01	90.46	88.46	54.78	73.61
			Precision (%)	71.02	90.93	88.23	64.08	71.51	77.15
*k*-NN	16	50	Sensitivity (%)	78.10	83.04	95.69	85.60	71.58	82.80
			Precision (%)	84.53	86.19	85.28	82.05	86.04	84.82
SVM	16	25	Sensitivity (%)	76.87	87.14	96.28	90.62	75.80	85.34
			Precision (%)	83.30	95.64	94.58	92.70	78.41	88.93

ADA: AdaBoost; DT: decision tree; RF: random forest; NB: Naïve Bayes; *k*-NN: *k*-nearest neighbors; SVM: support vector machine.

**Table 5:** The performance of different regression models for amount estimation (Unit: %).

Regression Model	Container-Independent		Container-Dependent							
			Can		Bottle		Handleless Mug		Handled Mug	
	MPE	MAPE	MPE	MAPE	MPE	MAPE	MPE	MAPE	MPE	MAPE
Linear	28.96	49.53	27.73	48.51	20.65	44.22	24.12	43.16	24.44	44.53
Gaussian	15.72	44.45	18.58	45.80	23.13	44.20	11.19	38.66	11.64	41.10
SVM-linear ^1^	12.68	40.06	5.65	29.53	9.65	34.28	7.14	38.94	16.28	46.69
SVM-Poly ^2^	24.51	47.75	24.97	47.49	22.24	45.45	19.26	41.14	19.91	42.74
SVM-RBF ^3^	20.66	45.93	18.86	44.25	14.01	38.92	14.93	40.86	19.96	45.80

^1^ SVM-linear: SVM regression model with linear kernel function; ^2^ SVM-Poly: SVM regression model with polynomial kernel function; ^3^ SVM-RBF: SVM regression model with RBF kernel function.

**Table 6:** The performance of different situations for amount estimation (Unit: %).

Situation	Container-Independent		Container-Dependent							
			Can		Bottle		Handleless Mug		Handled Mug	
	MPE	MAPE	MPE	MAPE	MPE	MPE	MAPE	MPE	MPE	MAPE
(1) Drinking Activity	−34.85	65.86	−27.24	51.59	−0.82	50.76	−35.89	69.09	−1.30	55.51
(2) Sip Gesture	−12.34	40.11	−29.09	47.28	−8.41	36.52	−5.90	40.77	−8.17	39.87
